# New Insights of Microsporidial Infection among Asymptomatic Aboriginal Population in Malaysia

**DOI:** 10.1371/journal.pone.0071870

**Published:** 2013-08-27

**Authors:** Tengku Shahrul Anuar, Hesham M. Al-Mekhlafi, Fatmah Md Salleh, Norhayati Moktar

**Affiliations:** 1 Department of Parasitology and Medical Entomology, Faculty of Medicine, Universiti Kebangsaan Malaysia, Kuala Lumpur, Malaysia; 2 Department of Parasitology, Faculty of Medicine, University of Malaya, Kuala Lumpur, Malaysia; Fundacion Huesped, Argentina

## Abstract

**Background:**

Studies on microsporidial infection mostly focus on immunodeficiency or immunosuppressive individuals. Therefore, this cross-sectional study describes the prevalence and risk factors of microsporidiosis among asymptomatic individuals in Malaysia.

**Methods/Findings:**

Four hundred and forty seven stool samples were collected and examined for microsporidia after staining with Gram-chromotrope Kinyoun. Demographic, socioeconomic, environmental, and behavioral information were collected by using a pre-tested questionnaire. Overall, 67 (15%) samples were positive for microsporidia. The prevalence of infection was significantly higher among individuals aged more than 15 years compared to those aged <15 years (OR = 1.97, 95% CI = 1.08, 3.62; *P* = 0.028). Furthermore, logistic regression analysis confirmed that the presence of other family members infected with microsporidia (OR = 8.45; 95% CI = 4.30, 16.62; *P*<0.001) and being a consumer of raw vegetables (OR = 2.05; 95% CI = 1.15, 3.66; *P* = 0.016) were the significant risk factors of this infection.

**Conclusions:**

These findings clearly show that exposure to microsporidia is common among Aboriginal population. Further studies using molecular approach on microsporidia isolates from asymptomatic individuals is needed to determine species-specific. The risk factors associated with microsporidiosis will help in identifying more clearly the sources of the infection in the environment that pose a risk for transmission so that preventive strategies can be implemented.

## Introduction

Microsporidia have been recognized as opportunistic pathogens of immunocompromised patients, but microsporidiosis is also becoming increasingly common in immunocompetent individuals [1]. Most human microsporidial infections are caused by *Enterocytozoon bieneusi* or *Encephalitozoon intestinalis* [2], [3]. *E. bieneusi* is found mainly in the upper gastrointestinal tract and is associated with chronic diarrhea and loss of weight, although a couple of reports have identified *E. bieneusi* in respiratory samples [4]. *E. intestinalis*, the second most frequently identified microsporidian causes disseminated microsporidiosis, affecting the gastrointestinal tract. On the other hand, *Encephalitozoon cuniculi* is an important parasite of domestic animals including rabbits, dogs, cats, cows, sheep and horses. Symptomatic human infections with this species are rare [5].

Intestinal microsporidia is very small (1 to 2 µm), single celled obligate intracellular parasites characterized by a polar filament that is extruded during the invasion of the host cell [6]. Mature microsporidia spores have thick three-layered walls, can pass through some water treatment filters due to their small size and are resistant to chlorine at concentrations used in treating drinking water. Microsporidia spores have been found in drinking water sources, soil, and domestic and wild animals; suggesting the possibility of water-borne, food-borne, zoonotic, and anthroponotic transmissions [7], [8].

Studies examining the prevalence of human microsporidiosis have been limited to patients who are positive for human immunodeficiency virus (HIV). However, recent molecular epidemiological studies have shown that organ transplant recipients and other immunocompromised patients, as well as immunocompetent individuals are at risk for infections that are mostly asymptomatic [9], [10], [11]. In contrast, only a few reports concerning microsporidial infection of immunocompetent individuals have been published [12], [13], [14], [15]. The most frequent clinical manifestations caused by microsporidia in AIDS patients are diarrhea, nausea, vomiting, malabsorption, and loss of weight. On the other hand, this infection usually causes self-limited diarrhea in immunocompetent individuals [16], [17],[18].

In Malaysia, few reports have been published regarding the prevalence of microsporidiosis among hospitalized patients, HIV infected patients, and Aboriginal communities [19], [20], [21], [22], [23]. However, to our knowledge, reports on the risk factors of microsporidial infection are lacking. Therefore, the present study aimed to determine the prevalence and associated risk factors of microsporidiosis among asymptomatic Aboriginal individuals in rural Malaysia. The establishment of such data will be beneficial for the public health authorities in the planning and implementation of specific prevention and control strategies of this infection in this population.

## Materials and Methods

### Study area and population surveyed

This cross-sectional study was conducted from June to December, 2011 among 447 Aboriginal participants living in eight villages from three different states (Pahang, Perak, and Negeri Sembilan) in suburban and remote areas of Peninsular Malaysia. The Aborignal is a collective term for a group of indigenous people that usually reside in the interior regions of Peninsular Malaysia. They identify themselves by tribes i.e. Proto-Malay, Negrito, and Senoi. They comprise about 0.6% of the total population in Malaysia. Sample selection was achieved using random selection of villages and random selection of 10 to 15 households per village. Within each village, participants over 2 years of age and those who provided consent to participate were included in this study. Exclusion criteria included children less than 2 years old and refusal to participate.

With regard to the age groups, 194 (43.4%) were less than 15 years old while 253 (56.6%) were 15 years old or more (≥15), with a median age of 20 years [interquartile range (IQR) 9–35]. Participants who participated in this study comprised of 197 (44.1%) males and 250 (55.9%) females.

### Sample size

With an expected prevalence of microsporidia among the Aboriginal population in Malaysia at 20% [22], [23], the 95% confidence interval (CI) and an absolute precision of 0.05 [24], the minimum sample size required for the study was estimated to be 246 participants.

### Questionnaire

A structured questionnaire was developed in English and then translated to Bahasa Melayu (the national language of Malaysia). The questionnaire was pre-tested among Aboriginal who was admitted to Gombak Hospital, Selangor state. Trained research assistants interviewed participants in person, asking questions for demographic data (i.e. age, gender and education level), socioeconomic background (i.e. occupation, household income, and educational status), behavioral risks (i.e. personal hygiene such as hand washing and food consumption), environmental sanitation and living condition characteristics (i.e. types of water supply, latrine system, sewage disposal system, and presence of domestic animals). Participants were also asked if they had diarrhea and symptoms of gastroenteritis (i.e. fever, vomiting, nausea, abdominal pain, watery stools, and blood or mucus stools). For children, the questionnaire was completed by interviewing their parents or the guardians who had given informed consent.

### Stool sample collection

Following the administration of the questionnaire, a wide mouth screw-capped container pre-labeled with the individual's name and code was distributed to each participant for the collection of a stool sample the next day. Their ability to recognize their name was counter-checked. The participant was instructed to scoop a thumb sized stool sample using a provided scoop into the container. Then, the container was placed in a zip-locked plastic bag. Parents and guardians were instructed to monitor their children during the sample collection in order to ensure that they placed their stool samples into the correct container. All participants were asked to provide sufficiently large stool sample (at least 10 grams).

### Detection of microsporidia by Gram-chromotrope Kinyoun staining

Thin stool smears were spread on glass slides; air dried, fixed with methanol, and stained with crystal violet for one minute and the excess stain was rinsed off with Gram's iodine. The slides were then stained with Gram's iodine for two minutes. The Gram's iodine solution was removed by gently rinsing with a decolorizer until the flow become colorless. The slides were washed with tap water and stained with chromotrope stained and prepared as described by Moura et al. [25] for eight minutes. The slides were rinsed in 90% acid-alcohol and counterstained with Kinyoun's carbol fuchsin for three minutes. Also, the slides were rinsed in 90% acid-alcohol, 95% alcohol for five minutes, and 100% ethyl alcohol for two minutes [20]. Then mounted with DPX medium (mixture of distyrene, plasticizer, and xylene) and covered with cover slips.

The criterion used to define microsporidia-positive was the presence of one or more pink-blue ovoid structures with a blue spore wall and a belt-like stripe encircling the spore, in at least 100 fields examined under 1000× magnifications, and confirmed by two technologists. The spore seen in the stained samples was graded as follows: 1+ (average number of spores seen was 1–10), 2+ (average number of spores seen was 11–20), and 3+ (average number of spores seen was >21) [19].

### Data management and analysis

Data was entered into a Microsoft Access and was cross-checked by the technical staff in order to ensure that data were entered correctly. Statistical analysis was performed using the SPSS version 20 (SPSS, Chicago, IL, USA). Only those participants who had Gram-chromotrope Kinyoun staining result together with complete questionnaire data were included in the final analyses.

For descriptive analysis, rate (percentage) was used to describe the characteristics of the studied population, including the prevalence of microsporidia. A Chi-squares test (χ^2^) was used to test the associations between the variables. In the univariate analysis, the dependent variable was prevalence of microsporidia, while the independent variables were demographic (gender and age group) and socioeconomic factors, behavioral risks, environmental sanitation, living condition characteristics, and gastrointestinal symptoms. All variables that were significantly associated with the prevalence of microsporidia in the univariate model were included in a logistic regression analysis to identify the risk factors for microsporidiosis with control for the effects of possible confounders. For each statistically significant factor, an odds ratio (OR) and 95% confidence interval (CI) were computed by the univariate and logistic regression analyses. The level of statistical significance was set as *P*<0.05.

### Ethical consideration

Ethical approval was obtained from the Ethics Committee of Universiti Kebangsaan Malaysia Medical Centre (UKMMC) (Reference Number: UKM 1.5.3.5/244/FF-165-2011) and permission for field work was obtained from the Ministry of Rural and Regional Development Malaysia before starting the study. Village meeting were held and village authorities and villagers were handed detailed explanations of the aims, procedures, potential risks, and benefits of the study. During the meeting, they were also informed that their identity and personal information would be kept strictly confidential and they could withdraw from the study at any point of time without citing reasons for doing so. If they agreed to participate, their consent was obtained in written form (signature or thumbprint for those who were illiterate). For children, written informed consent was obtained from their parents. Ethics committee has also approved the consent procedure used in this study.

## Results

### Characteristics of the study population

Single stool samples were randomly collected from a total of 447 participants. About two thirds (65%) of the parents have low level of education i.e. less than 6 years of formal education. Most of the parents did odd jobs, such as selling forest products without any stable income. Some were daily wage earners working in rubber or palm oil plantations, unskilled laborers in factories or on construction sites. Therefore, 47.8% of the households belonged to people who earned less than RM500 per month (US$162.42). Although 60.9% of the houses have a provision of basic infrastructure such as treated water supply and 66% have a pour flush toilet, at least 39.1% are still using untreated water originating from a nearby river for their domestic needs and 34% still defecate indiscriminately in the river or bush. More than half (55.7%) of the households kept dogs, cats, and poultry as their domestic animals.

### Prevalence of microsporidial infection

**Figure 1:**
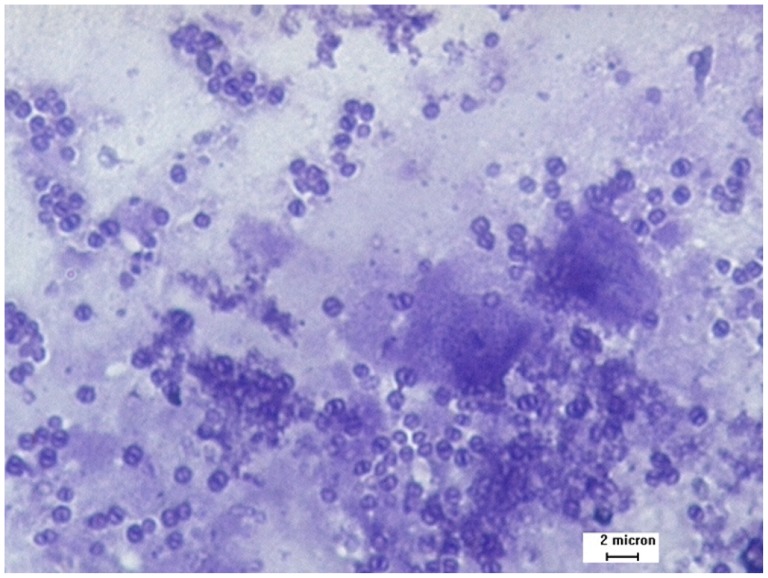
Microsporidia spores from stool samples stained with Gram-chromotrope Kinyoun under light microscopy (magnification, ×1000). Clear background and the polar tubes of almost all spores were stained.

Table 1 shows that 15% (67/447) of the participants were infected with microsporidia. Of the 67 positive smears, 65 (97%) had low spore counts of 1–10 per 100 fields/100×, while only 2 (3%) had moderate spore counts of 11–20 per fields/100× (Figure 1). The prevalence of microsporidial infection was not statistically significant between genders. However, this infection was significantly higher in the age group more than 15 years old.

**Table 1:** Prevalence of microsporidial infection among asymptomatic Aboriginal according to age groups and gender.

	Orang Asli population		
	*n* positive	*n* examined	% positive
**Age groups (years)**			
<15	20	194	10.3
≥15	47	253	18.6
**Gender**			
Male	30	197	15.2
Female	37	250	14.8
**Total**	67	447	15.0

*n* = Number examined.

### Associated risk factors for microsporidiosis

The association of microsporidial infection and sociodemographic characteristics are shown in Table 2. The results of univariate analysis showed that the prevalence of microsporidiosis was significantly higher among individuals aged more than 15 years (18.6%; 95% CI = 1.13, 3.48) when compared with those aged <15 years. Similarly, the prevalence was significantly higher among participants who consume raw vegetables (19.4%; 95% CI = 1.15, 3.36) and those children of mothers with less than 6 years of formal education (16.9%; 95% CI = 1.42, 12.21). Moreover, the presence of other family members infected with microsporidia increased children's odds for infection when compared with their counterparts by 7.4 times (95% CI = 3.89, 14.18)

**Table 2:** Potential risk factors associated with microsporidial infection among asymptomatic Aboriginal population (univariate analysis, *n* = 447).

Variables	No. examined	Infected (%)	OR (95% CI)	*P*-value
**Age (years)**				
≥15	253	18.6	1.99 (1.13,3.48)	0.015^a^
<15	194	10.3	1	
**Gender**				
Male	197	15.2	1.03 (0.61,1.74)	0.900
Female	250	14.8	1	
**Drinking untreated water**				
Yes	175	16.6	1.22 (0.72,2.07)	0.452
No	272	14.0	1	
**Bathing and washing in the river**				
Yes	130	16.9	1.23 (0.71,2.15)	0.463
No	317	14.2	1	
**Not washing hands after playing with soil or gardening**				
Yes	157	13.4	0.82 (0.47,1.43)	0.482
No	290	15.9	1	
**Presence of domestic animals**				
Yes	198	16.2	1.18 (0.70,1.98)	0.536
No	249	14.1	1	
**Indiscriminate defecation**				
Yes	152	17.1	1.28 (0.75,2.18)	0.368
No	295	13.9	1	
**Sewage disposal**				
Outdoor	197	16.8	1.28 (0.76,2.15)	0.354
Common drainage	250	13.6	1	
**Eating with hands**				
Yes	288	17.0	1.61 (0.90,2.87)	0.106
No	159	11.3	1	
**Consuming raw vegetables**				
Yes	217	19.4	1.97 (1.15,3.36)	0.012^a^
No	230	10.9	1	
**Eating fresh fruits**				
Yes	347	15.9	1.38 (0.71,2.70)	0.342
No	100	12.0	1	
**Father's education**				
Non-educated (<6 yrs)	180	14.4	1.60 (0.69,3.71)	0.266
Educated (≥6 yrs)	84	9.5	1	
**Mother's education**				
Non-educated (<6 yrs)	178	16.9	4.16 (1.42,12.21)	0.006^a^
Educated (≥6 yrs)	86	4.7	1	
**Working mothers**				
Yes	112	12.5	0.94 (0.45,1.96)	0.875
No	152	13.2	1	
**Household members**				
≥8 (large)	162	19.1	1.64 (0.97,2.77)	0.064
<8	285	12.6	1	
**Household monthly income**				
≤RM500 (low)	213	16.0	1.16 (0.69,1.95)	0.582
>RM500	234	14.1	1	
**Other family members infected with microsporidia**				
Yes	48	47.9	7.42 (3.89,14.18)	<0.001^a^
No	399	11.0	1	

RM = Malaysian Ringgit; (US$100 = RM307.85) [1^st^ February 2013].

Reference group marked as OR = 1 [OR = Odds Ratio].

CI = Confidence interval.

a Significant association (*P*<0.05).

Multiple logistic regression analysis confirmed that being aged more than 15 years (OR = 1.97, 95% CI = 1.08, 3.62; *P* = 0.028), consuming raw vegetables (OR = 2.05; 95% CI = 1.15, 3.66; *P* = 0.016), and the presence of other family members infected with microsporidia (OR = 8.45; 95% CI = 4.30, 16.62; *P*<0.001) as the significant risk factors for microsporidiosis in the population studied (Table 3).

**Table 3:** Logistic regression analysis of risk factors associated with microsporidiosis among asymptomatic Aboriginal population.

Variables	OR	95% CI	*P*-value
Being aged more than 15 years	1.97	1.08, 3.62	0.028
Consuming raw vegetables	2.05	1.15, 3.66	0.016
Presence of other family members infected with microsporidia	8.45	4.30, 16.62	<0.001

### Co-infection with other intestinal parasites

For all positive stool samples with microsporidia (*n* = 67), 79.1% showed co-infection with one or more other parasites. *Trichuris trichiura* was the most common intestinal parasite found in conjunction with microsporidia (58.2%), followed by *Entamoeba histolytica/dispar/moshkovskii* (25.4%), *Entamoeba coli* (19.4%), *Giardia intestinalis* (13.4%), *Blastocystis hominis* (13.4%), *Ascaris lumbricoides* (13.4%), hookworm (10.5%), *Iodamoeba buetschlii* (7.5%), and *Entamoeba hartmanni* and *Chilomastix mesnilii* (4.5%).

## Discussion

Microsporidiosis in humans occurs worldwide but prevalence vary widely depending on the geographical region, population studied and diagnostic methods used [26], [27]. Prior to the AIDS pandemic, microsporidiosis was rarely identified in humans [28], [29]. Prevalence data for microsporidia in human populations before the era of AIDS relied upon serology based on detecting antibodies to *Enterocytozoon bieneusi*, *Encephalitozoon intestinalis* or *Encephalitozoon cuniculi*, the species that isolated in mammals. Seroprevalence results ranged from 0 to 42%, with the highest rates found in homosexual men in Sweden and in persons with other parasitic infections [30], [31]. A wide range of case reports have identified microsporidia in non-HIV infected individuals but prevalence data based on parasite detection (versus serology) are lacking [32]. The prevalence in non-HIV infected individuals have been reported using microscope studies and polymerase chain reaction (PCR) technique in travelers to endemic areas (3.3 to 10%), children with diarrhea or not (1.7 to 17.4%), and elderly peoples (17.2%) [1], [33], [34].

In this study, the prevalence of microsporidia among the asymptomatic Aboriginal in Malaysia was 15%, which differs from previous local reports [21], [22], [23]. Comparing our findings with studies from other countries showed that the prevalence reported by the present study was similar to those reported in Spain (17%), Uganda (16.8%), Thailand (14.9%), and Czech Republic (15%) [1], [10], [17], [35]. By contrast, considerably lower prevalence rates of 0.7%, 8%, 5.35% and 9.3% were reported in Germany, Peru, Tunisia, and Nigeria among immunocompetent individuals, respectively [36], [37], [38], [39]. The prevalence of microsporidia in the asymptomatic Aboriginal was significantly higher than other groups of Malaysian population and it is in agreement with the earlier assertion that intestinal parasitic infection, in general among the Aboriginal community is higher than in the general healthy population in Malaysia [21], [40]. Tapioca is the main food group for the people in this study and the villagers forage in the jungle for additional sustenance, where there is a risk of acquiring microsporidial infection, owing to the parasite's ubiquity in nature [41]. Moreover, it is possible that low immune status among this people due to the high prevalence of protein-energy malnutrition make them more susceptible to microsporidial infection, as there appears to be a relationship between malnutrition and other enteric parasitic infection in this population [21], [42]. Furthermore, shedding of microsporidia spores is intermittent and it is known that patients diagnosed with microsporidiosis by analysis of biopsy material may have stools devoid of the parasite [43]. Thus, the prevalence of microsporidia among asymptomatic Aboriginal in Malaysia would be expected to be even higher than the analysis of stool indicates.

The present findings showed no significant difference in the prevalence of microsporidia according to gender of the participants, and this is consistent with the results of previous reports [22], [23], [35], [44]. On the other hand, the current study reported significantly higher odds of microsporidial infection among those aged more than 15 years as compared to younger individuals. Likewise, a recent parasitological evaluation of stool samples from Czech Republic citizens (immunocompetent individuals and foreign students of varying age groups) had identified 34 to 56% prevalence rates of shedding *E. bieneusi* and *Encephalitozoon* species with the highest prevalence in the group of 50 years and above [35]. Similarly, Norhayati et al. [19] also reported a high prevalence (57.2%) of microsporidiosis among adults aged more than 31 years. The high prevalence rate of the infection among those aged ≥15 years in the present study might be explained by the fact that their behavior is related to more active movement and more independent eating habits compared with children. However, it is also possible that microsporidia persisted but was shed less consistently or at levels below detection in younger age group.

Few studies have been conducted to determine the source and mode of transmission of intestinal microsporidiosis. Information from individual case reports of microsporidiosis in humans and from both natural and experimental infections in animals however has provided insights about the likely modes of transmission of microsporidia to humans [45], [46]. The observations that microsporidia are ubiquitous in nature suggest that several modes of transmission and sources exist for human infections.

The possibility of human-to-human transmission cannot be ruled out. Experimental examples of microsporidia transmission between laboratory animals might suggest that the same infecting species could be transmitted between humans via the same pathways as in animals [34], [47]. On further analysis of risk factor using multiple logistic regression analysis, we found that the presence of other family members infected with microsporidia increases the odds for acquiring microsporidiosis by 8 times. Likewise, a study done by Leelayoova et al. [48] suggested that human-to-human transmission occurred in an orphanage, where a multivariate analysis showed that orphans who were 12–23 months old and living in one particular house were independently associated with *E. bieneusi* infection and all infected children presented the same genotype in a stool samples. Few studies had also provided evidence to support the role of human-to-human transmission in intestinal microsporidiosis. Individuals with a history of contact with diarrheal patients had two times greater risk of getting the microsporidial infection [49].

Microsporidia are among the parasites of concern for food-borne transmission as a result of globalization of food, faster transport of food, increasing travel of consumers, and changes in food consumption patterns [50], [51]. It is interesting to note that in this study, those who consumed raw vegetables were at 2 times odds of being infected with microsporidia when compared to those who consumed cooked vegetables. Similarly, Calvo et al. [52] identified spores of microsporidia on lettuce, parsley, cilantro, and strawberries in Costa Rica. Furthermore, a recent study conducted by Decraene et al. [53] suggested that cucumber slices in both cheese sandwiches and a salad were the most probable vehicle of transmission for food-borne outbreak associated with *E. bieneusi* in Sweden. Washing of fresh product such as beans and lettuce remains of utmost important in preventing food-borne illness and should continue to be emphasized. Sometimes, washing may be insufficient to remove all pathogens including microsporidia spores. In this instance, it may be postulated that the level of contamination was quite high that washing procedure was unable to remove enough of the pathogen load, so as to prevent infection. Alternatively, it may be that microsporidia spores are capable of strong adhesion to or internalization in, certain types of product, thereby successfully evading the effects of washing and disinfection. A research conducted in the USA demonstrated that *Cryptosporidium* oocysts were capable of strongly adhering to spinach plants after contact with contaminated water and were also internalized within the leaves, thus making washing entirely ineffective [54].

Several questions still exist about clinical aspects and consequences of microsporidial infection in humans. In general, the clinical course of microsporidiosis depends on the immune status of the host and site of infection [6]. Immunocompromised hosts infected with microsporidia were most likely to develop disease that often contributed to death [55], [56]. In healthy humans such as travelers, self-limiting diarrhea with duration of about 2–3 weeks has been reported [36], [57]. Furthermore, a study conducted in Czech Republic showed only a minority of diarrheal samples was positive with microsporidia and it was present mainly in formed stool samples [35]. Therefore, it seems important to determine if asymptomatic and persistent microsporidial infection occur in humans, and if so, improved and reliable diagnostic methods are needed for attempting to prevent transmission to others at risk or to reduce the potential for reactivation of infection.

Several limitations apply to our findings. Firstly, identification of microsporidia at species level cannot be achieved by current standard light microscope technique. Transmission electron microscope (TEM) or polymerase chain reaction (PCR) is required to achieve this task. Hence, the findings are presented as microsporidial infection without mention of the species. However, previous study carried out in the same setting reported *E. bieneusi* and *E. intestinalis* were the common microsporidia species isolated [58]. Furthermore, the detection limit of light microscope has been determined to be between 104 and 106 microsporidia spores per gram of stool, whereas PCR is able to detect spore concentrations as low as 102 per grams of stool [59], [60]. Thus, the true prevalence of microsporidia has to be determined by highly sensitive techniques such as PCR. Secondly, stool samples may contain spores of non-human microsporidia which acquired from insects, crustacean or fish that can passed through the digestive system. These spores would stain similarly as human microsporidia. Therefore, in order to overcome this limitation, Gram-chromotrope Kinyoun staining was used for identification of microsporidia spores. The staining has higher sensitivity and specificity of 98% and 98.3%, respectively as compared to the reference technique Weber modified trichrome with statistically significant agreement by Kappa statistics (K = 0.941; *P*<0.001) [20]. Furthermore, morphological characteristics and spore size were used for the identification in the present study. Using the previous staining technique described by Fatmah et al. [20], the wall of each spore was stained blue and the polar tube stained deep blue in color, encircled each spore. The size of each microsporidia spore was approximately 1.5 by 0.9 µm. On the other hand, yeast cells were distinguished by their larger size (3 µm). Bacteria stained faintly with this stain and could not be easily confused with microporida spore. Moreover, *Nosema locustae* (insects microsporidia) and *Pleistophora* spp. (arthropods microsporidia) were 2.0 by 3.0 µm and 2.8 by 4 µm in size, respectively [61]. Finally, the unavailability of information on immune status of the participants, therefore, the cause-effect association could not be determined in this study.

In conclusion, the data obtained during the present study showed high prevalence of this infection among the asymptomatic Aboriginal population, raising the question of whether the findings represent true infection resulting in shedding of parasites or ingested parasites that just passed through the gastrointestinal tract without establishing an infection. Since no data exist on the risk factors among this population, our findings on the risk factors associated with microsporidiosis will help in identifying more clearly the sources of the infection in the environment that pose a risk for transmission so that preventive strategies can be implemented. Further studies of people living in different parts of the country also should be performed. Moreover, genotyping of microsporidia isolated from asymptomatic individuals might determine whether there are microsporidia of various levels of virulence.
